# Christian Jassoy, Jens-Karl Eilers, Andreas Sönnichsen: Wissenschaftskompetenz in der Medizin

**DOI:** 10.3205/zma001657

**Published:** 2024-02-15

**Authors:** Volker Paulmann

**Affiliations:** 1 Medizinische Hochschule Hannover, Studiendekanat, Bereich Lehr- und Lernforschung, Hannover, Germany

## Bibliographical details

Christian Jassoy, Jens-Karl Eilers, Andreas Sönnichsen

**Wissenschaftskompetenz in der Medizin**

Publisher: Thieme

Year of publication: 2022, 184 pages, price: € 39.99

ISBN: 978-3-13-243209-3

## Review

In recent years, new teaching concepts focused on imparting scientific competencies have emerged at many medical faculties. However, there is a glaring lack of accompanying literature that systematically addresses the specific challenges in the field of medicine related to these learning and teaching processes. So far, medical students, primarily during their doctoral phases, have had to resort to scattered scripts or literature from outside the subject. With the approximately 180-page book publication authored by Jassoy/Eilers/Sönnichsen, according to the publisher's information, the “first textbook on the new subject (sic!) Scientific Competence” is now available. In principle, however, the target audience can encompass anyone who want to deal with scientific issues within the field of medicine. In line with its textbook character, sections or subchapters can be selectively read without losing orientation. Cross-references and citations allow for deeper exploration within the book or in further literature. The index also facilitates quick access.

The first section, spanning approximately 30 pages, delves into the fundamental research paradigms of medicine (observations and hypotheses) and the common study and reporting formats, ranging from basic experimental research to clinical, diagnostic, epidemiological questions, and healthcare research.

The second section explains basic statistical concepts and introduces the main tests and their application areas.

The third section focuses on the practical implementation of studies. It covers the process of knowledge acquisition, from information gathering and project planning, including sample design, to the danger of bias and ethical aspects.

The last and most extensive part of the book is dedicated to the central goals, methods, and evaluation steps of evidence-based medicine (EBM). Embedded in the presentation of systematised research strategies, the scientific methods are brought together here at the level of patient care. This sets a central point in the development of scientifically competent physicians – the ability to independently seek complex information, evaluate it scientifically, and apply it in the clinical context for the benefit of patients.

The four main chapters share the aim of comprehensively incorporating the relevant aspects of scientific work in medicine, which is convincingly achieved. However, the “dynamic” developments in the scientific field are sometimes marginalized during this mapping process. For instance, in the use of animal models (p. 96f.), it would be possible to illustrate the problems and alternatives currently being discussed within the scientific community. Assessments of future developments would also be worth reading for the aberrations (and new approaches) in publishing, not least against the background of artificial intelligence.

Regarding the book’s design, there is a clear effort to address a student audience and their learning habits. In the two-column layout with small font size, short narrative text, informative graphics, and tables alternate. At the end of the compressed, sometimes glossary-like sections, the most important content is summarized once again in color-coded “Key Boxes”. The authors rarely deviate from sober textbook prose, except in the sections marked as “in-depth knowledge”, which incorporate experiential knowledge and unwritten rules of the scientific field. For some of the presented phenomena, such as the prevalence of “predatory journals” (p. 109), concrete advice on appropriate handling would have been desirable. A stronger orientating hand would certainly also offer added value in the basic statistical section. At least for the most common tests (table 16.2, p. 67), references to further explanatory literature would certainly be helpful in facilitating the practical steps in one's scientific work.

### Conclusion

In view of the requirements that the National Competence-Based Catalogue of Learning Objectives in Medicine (NKLM) and the new licensing regulations place on scientific work in medical studies, the book offers a valuable orientation framework. Its strength undoubtedly lies in the breadth of the considered aspects, which comprehensively define the requirement profile of scientific literacy. It is hardly avoidable that in-depth exploration of specific aspects occasionally falls by the wayside. However, it makes it clear to readers that scientific thinking and acting are fundamental to medical practice and must be firmly integrated into studies and training. It would have been desirable to occasionally compare medicine with other disciplinary cultures, which could sharpen the perception of the universality as well as the idiosyncrasies of medical science, especially for young medical professionals. In addition to the brief introductory excursion into the history of medicine, an epistemological perspective would have been helpful: Is medicine as a scientific discipline only to be understood as a sum of individual subjects (natural sciences, social sciences, canon of methods, etc.)? And what, in contrast, might the characteristics of specific “scientific competence in medicine” be?

From a didactic perspective, an examination of the relationship between research and teaching in medicine would have been enriching. In the array of presented medical research areas in the first section, medical education research is unfortunately given only three brief sentences. Nevertheless, for curriculum development, the book offers various starting points for imparting scientific competencies, especially in identifying one’s own blind spots.

### Outlook

The changes in the research landscape, the immeasurably growing flood of publications, the incomprehensible possibilities of artificial intelligence, as well as the evolving relationship between science and the public demand a keen eye for new trends from the young generation of researchers. In this respect, there are still numerous opportunities – for the textbook and other publications – to be linked to and deepened.

## Note

The English version of the review was created with the help of DeepL and ChatGPT.

## Author’s ORCID

Volker Paulmann: 0000-0002-9143-2794

## Competing interests

The author declares that he has no competing interests.

